# Insights into the Structure of Comirnaty Covid-19 Vaccine: A Theory on Soft, Partially Bilayer-Covered Nanoparticles with Hydrogen Bond-Stabilized mRNA–Lipid Complexes

**DOI:** 10.1021/acsnano.2c11904

**Published:** 2023-07-07

**Authors:** János Szebeni, Bálint Kiss, Tamás Bozó, Keren Turjeman, Yael Levi-Kalisman, Yechezkel Barenholz, Miklós Kellermayer

**Affiliations:** † Nanomedicine Research and Education Center, Department of Translational Medicine, Semmelweis University, Budapest 1089, Hungary; ‡ Department of Nanobiotechnology and Regenerative Medicine, Faculty of Health Sciences, Miskolc University, Miskolc 2880, Hungary; Ψ School of Chemical Engineering and Translational Nanobioscience Research Center, Sungkyunkwan University, Suwon 16419, Korea; ξ Department of Biophysics and Radiation Biology, Semmelweis University, Budapest 1094, Hungary; + Hungarian Centre of Excellence for Molecular Medicine (HCEMM), In Vivo Imaging Advanced Core Facility, Budapest 1094, Hungary; ⊄ ELKH-SE Biophysical Virology Research Group, Budapest 1094, Hungary; ⊃ The Laboratory of Membrane and Liposome Research, IMRIC, Hebrew University-Hadassah Medical School, Jerusalem 9112102, Israel; ≡ Institute of Life Sciences and the Center for Nanoscience and Nanotechnology, The Hebrew University of Jerusalem, Edmond J. Safra Campus, Givat Ram, Jerusalem 9190401, Israel

**Keywords:** lipid nanoparticles, phospholipid membranes, Doxil liposomes, SARS-CoV-2, atomic force microscopy, cryo-electron microscopy, dynamic light scattering

## Abstract

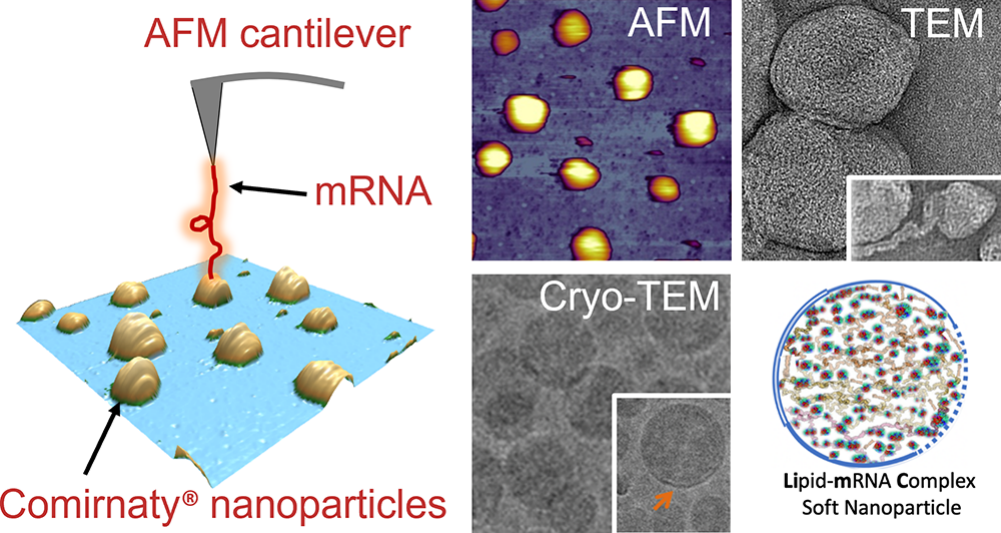

Despite the worldwide success of mRNA-LNP Covid-19 vaccines, the nanoscale structures of these formulations are still poorly understood. To fill this gap, we used a combination of atomic force microscopy (AFM), dynamic light scattering (DLS), transmission electron microscopy (TEM), cryogenic transmission electron microscopy (cryo-TEM), and the determination of the intra-LNP pH gradient to analyze the nanoparticles (NPs) in BNT162b2 (Comirnaty), comparing it with the well-characterized PEGylated liposomal doxorubicin (Doxil). Comirnaty NPs had similar size and envelope lipid composition to Doxil; however, unlike Doxil liposomes, wherein the stable ammonium and pH gradient enables accumulation of ^14^C-methylamine in the intraliposomal aqueous phase, Comirnaty LNPs lack such pH gradient in spite of the fact that the pH 4, at which LNPs are prepared, is raised to pH 7.2 after loading of the mRNA. Mechanical manipulation of Comirnaty NPs with AFM revealed soft, compliant structures. The sawtooth-like force transitions seen during cantilever retraction imply that molecular strands, corresponding to mRNA, can be pulled out of NPs, and the process is accompanied by stepwise rupture of mRNA–lipid bonds. Unlike Doxil, cryo-TEM of Comirnaty NPs revealed a granular, solid core enclosed by mono- and bilipid layers. Negative staining TEM shows 2–5 nm electron-dense spots in the LNP’s interior that are aligned into strings, semicircles, or labyrinth-like networks, which may imply cross-link-stabilized RNA fragments. The neutral intra-LNP core questions the dominance of ionic interactions holding together this scaffold, raising the possibility of hydrogen bonding between mRNA and the lipids. Such interaction, described previously for another mRNA/lipid complex, is consistent with the steric structure of the ionizable lipid in Comirnaty, ALC-0315, displaying free =O and −OH groups. It is hypothesized that the latter groups can get into steric positions that enable hydrogen bonding with the nitrogenous bases in the mRNA. These structural features of mRNA-LNP may be important for the vaccine’s activities in vivo.

The worldwide use of Pfizer/BioNTech’s Comirnaty (BNT162b2) and Moderna’s Spikevax (mRNA-1273) vaccines against Covid-19 has brought substantial public and scientific interest and scrutiny. This approach of immunization is based on mRNA-containing lipid nanoparticles (mRNA-LNPs), wherein the mRNA encodes the virus’s spike protein (S-protein).^1,2^ Following translation, the protein presents an antigen for the immune system to develop specific immunity against the virus. This approach of vaccination stems from the success of LNPs in gene therapy and lipophilic drug delivery that utilizes ionizable lipids displaying positive charge at low pH (IPC lipids), neutral PEGylated lipids, neutral membrane-forming phospholipids, and cholesterol.^3−6^ These compact nanoparticles (NPs) are very different from the clinically applied bilayer liposomes with a clearly discernible internal aqueous compartment. Yet, there is little information about the molecular buildup of fully filled LNPs in general and Comirnaty, in particular. Accordingly, the goal of the present study was to employ a combination of state-of-the-art nanostructure analysis techniques to better understand the structure of Comirnaty in comparison with Doxil, a well-characterized liposome control, whose chemical composition is compared to that of Comirnaty in Supplement Table 1. We focus on the structure of vaccine NPs as administered to people, also noting changes observed after 1 day storage of diluted stock at 4 °C, which is irrelevant regarding the human application of Comirnaty but reveals information that helps understand the structure of these NPs.

We used atomic force microscopy (AFM) imaging and force spectroscopy to unveil the 3D shape and nanomechanical properties of Comirnaty, dynamic light scattering (DLS) to ascertain the averaged hydrodynamic size of NPs, cryogenic-transmission electron microscopy (cryo-TEM) to image the individual LNPs, their size and shape, and internal fine structure, and measurements of radiolabeled methylamine distribution (between intra- and extra-NP medium) to explore the presence of a transmembrane pH gradient. Our results, taken together with a recent description of IPC lipid clusters binding to mRNA with hydrogen bonds,^7^ led us to propose a model of Comirnaty structure wherein the apolar lipid core of the LNP and the highly convoluted mRNA strands are meta-stabilized by hydrogen bonds with IPC-lipids with weaker or absent ionic interactions and intermittent mono- and bilayered membranes making up the coat.

## Results/Discussion

### AFM Images of Comirnaty in the Form That Is Administered to People and after Storage of Expired Samples

**Figure 1:**
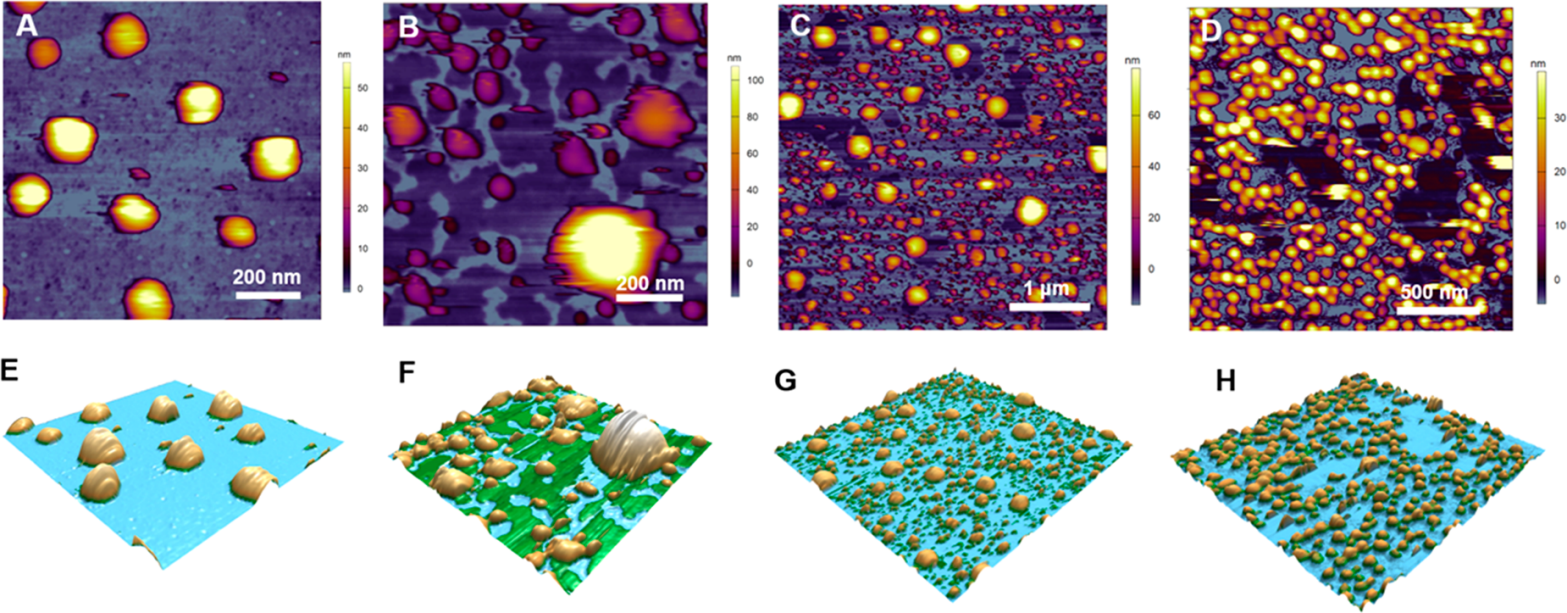
AFM height-contrast images (A–D) and corresponding 3D reconstructions (E–H) of Comirnaty vaccine and Doxil immobilized on a glass surface. (A) and (E) show a freshly diluted Comirnaty sample representing the jab inoculated into the deltoid muscle; (B) and (F) show a 1-day-old sample stored at 4 °C, representing the unused leftover; (C) and (G) show a refrozen sample, representing unintended acceleration of fragmentation by refreezing the leftover vaccine; (D) and (H) show a freshly opened Doxil sample.

Based on a preliminary study evaluating the optimal substrate for sample support (e.g., mica, glass, poly-l-lysine, or anti-PEG coated mica), we found clean glass as the best to immobilize the NPs for imaging. Accordingly, the AFM images of Comirnaty and Doxil NPs, under different conditions with regard to storage time and temperature, were prepared this way and shown in Figure 1.

It is seen in Figure 1, particularly in the 3D reconstituted versions of images (lower panels), that the freshly diluted vaccine (Figure 1A, E), representing the inoculum used for injection into humans, consists of monodisperse, spherical NPs of about 120–150 nm apparent diameter and 40–60 nm topographical height (54.6 ± 19.3 nm, mean ± SD, *n* = 148), corresponding to slightly flattened surface-adsorbed NPs. One-day storage of the diluted vaccine at 4 °C, which represents samples not recommended for human use, led to striking differences in sample morphology (Figure 1B, F). Namely, both the size and shape of NPs became heterogeneous, and large flat patches were visible on the supporting surface. Refreezing the stored samples, which also is excluded by the manufacturer from human use, led to even more heterogeneous, dispersed NPs with a substantial variety of fragment shapes in the ∼5 to ∼300 nm range (Figure 1C, G). The freshly opened Doxil (Figure 1D, H) showed monodisperse, spherical NPs similar to the freshly suspended Comirnaty NPs (Figure 1A, E), but their topographical height was lower (28.5 ± 7.8 nm, mean ± SD).

Regarding the morphological changes of Comirnaty upon storage, as shown by AFM, the increase of particle size without segmentation and loss of smooth surface may be explained by time-dependent association of NPs, most likely via fusion. The decrease of particle size, in turn, suggests that loss of phospholipids has occurred in some liposomes, giving rise to flat patches in the background that correspond to lipid layers stretched on the surface (Figure 1B, F).

It should be noted that a recent study, also using AFM to analyze the morphology of mRNA-LNPs mimicking the lipid composition of Comirnaty and Spikevax, showed the predominant presence of spherical and bleb-like NPs with a diameter < 60 nm. However, unlike in our study, the self-made NPs were immobilized by anti-PEG antibodies linked to protein G-covered glass.^8^ These differences highlight the uniqueness of each NP formulation and consequent need to identify appropriate methods for their visualization by AFM.

### DLS Analysis of the Hydrodynamic Size and Size Distribution of Freshly Diluted Comirnaty and Doxil

As shown in Table 1, the freshly diluted Comirnaty and Doxil NPs had essentially similar hydrodynamic sizes (diameter: 80–85 nm), but the homogeneity of Comirnaty was slightly lower than that of Doxil, as reflected in the higher span of size distribution and higher polydispersity index (PDI) of Comirnaty NPs compared to Doxil. Also, the surface charge (Zp) of Doxil (−31 ± 5.1 (mean ± SD, *n* = 3) was more negative than that of Comirnaty (8.6 ±5.3, mean ± SD, *n* = 3), as determined in the low ionic strength medium of 1.5 mM sodium nitrate. Nevertheless, these data are consistent with the AFM images inasmuch as both preparations consist of spherical NPs of about the same size. As for the discrepancy between the hydrodynamic diameter measured by DLS and topographical height of NPs, measured by AFM, it can be attributed to the different focuses of the two methods: global estimate of NP size in solution vs 3D shape of individual NPs adhered to a surface, respectively.

**Table 1:** DLS Analysis of the Hydrodynamic Size and Size Distribution of NPs in Freshly Diluted Comirnaty and Doxil^a^

NPs	Z-Ave	*D*(v) 10	*D*(v) 50	*D*(v) 90	*D*(i) 10	*D*(i) 50	*D*(i) 90	SPAN (i)	PDI	Zp
Comirnaty	84.4 ± 0.3	40.6 ± 0.9	61.2 ± 0.9	109 ± 1.7	53 ± 0.3	88.7 ± 1	155.3 ± 0.6	1.1	0.20	–8.6 ± 2.6
Doxil	81.5 ± 0.4	48.7 ± 1.0	68.9 ± 1.0	105 ± 0.6	58 ± 0.9	84.5 ± 0.3	123 ± 0.6	0.8	0.06	–31 ± 5.1

a Entries are mean ± SD, *n* = 3 measurements. *D*(v) and *D*(i) refer to the distribution percentiles of the same sample based on intensity (*D*i) or volume (*D*v) analysis. Span is defined as *D*_90_ – *D*_10_/*D*_50_ where *D*_10, 50 and 90_ are the percentiles under the size distribution curve; PDI, polydispersity index. Further details are described in the Methods section.

### Nanomechanical Properties of Freshly Diluted Comirnaty NPs

**Figure 2:**
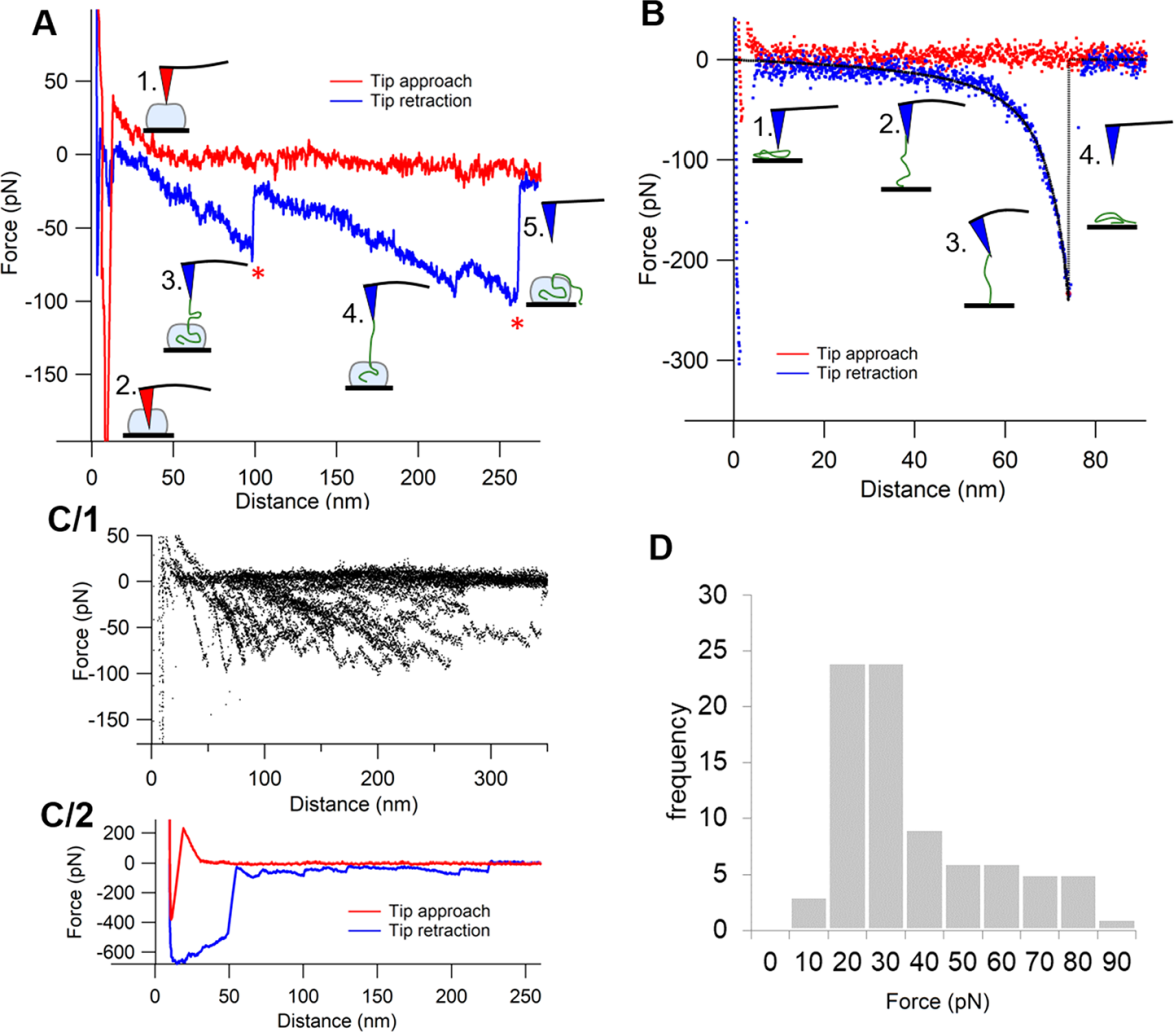
Force spectroscopy of Comirnaty. (A) Force–distance curve of a particle indentation (red curve) and RNA extraction (blue curve). The numbered schematics along the curves illustrate the different stages of measurements, with the red and blue tips pointing to the peak “force points”, i.e., the distance where sudden transitions occur. (B) Force–distance curve of RNA stretching and its related schematics. Black dashed line shows worm-like chain model fit (persistence length = 345 pm; contour length = 83 nm). (C/1) Region of interest of RNA extraction force–distance curves superimposed (*n* = 20). (C/2) A selected RNA extraction trial with multiple RNA structural transition events. (D) Histogram of peak forces detected in RNA extraction curves (peak forces are labeled with red stars in (A)).

To explore the nanomechanical properties of vaccine NPs administered to humans, we analyzed the force–distance curves obtained in freshly diluted Comirnaty samples upon NP indentation with the AFM’s cantilever. Figure 2A shows a representative force curve recorded during an indentation cycle. During tip approach (red curve), following a constant-force region reflecting the lack of load as the cantilever moved toward the NP surface, a rise in force was apparent (phase 1). The distance (ca. 50 nm) at which the force began to rise corresponds well to the topographical height of NPs, indicating the point of contact between the surface-adsorbed particle and the AFM tip. The apparent linear increase of force is a clear sign of elastic compression of the NP. The slope of the linear fit in this region provided a stiffness of ∼9 pN/nm (Table 2), which is slightly smaller than that of dimyristoyl-phosphatidylcholine (DMPC) liposomes^9^ and an order of magnitude smaller than that of dipalmitoyl-phosphatidylcholine (DPPC) liposomes^10^ with roughly the same radius (i.e., liquid-disordered and liquid-ordered membrane liposomes at room temperature, respectively).

**Table 2:** Nanoparticle Biomechanical Properties Obtained from AFM Image and Force Curve Analysis of Freshly Diluted Comirnaty^a^

parameter	Comirnaty
vesicle stiffness (pN/nm)	8.92 ± 8.25 (20)
rupture force (pN)	77.8 ± 47.2 (20)
attractive force (pN)	414.4 ± 276.9 (20)
peak force (pN)	42.7 ± 20.4 (83)

a Entries are mean ± SD from (*n*) tests. Further details are in the Methods.

At a certain distance, a sudden and considerable force drop leading to high negative force values occurred (phase 2), indicating that the particle surface ruptured and resulted in tip attraction. The distance at which rupture took place was ∼40% of the contact-point distance (i.e., contact height), suggesting that the particles could be compressed to almost 1/3 of their height during indentation before they lost their mechanical integrity. The force necessary to pierce through the NP surface (∼78 pN, Table 2) was 1–3 orders of magnitude lower than that in other ordered nanoscale biomolecular systems such as liposomes (0.6–1.1 nN),^9,10^ with roughly the same radius (i.e., liquid empty viral capsids (0.6–5.8 nN).^11−15^

The drop in force is not the usual phenomenon. Puncturing lipid vesicles normally leads to a much smaller force drop, leaving the force acting on the tip in the repulsive (positive) regime.^10,16,17^ This is due to the line tension of the lipid bilayer hole created by the tip. By contrast, the sudden, transitionless force drop observed for Comirnaty particles is caused by a quick and strong attractive interaction between the vesicle core and the silicon tip, suggesting that the LNPs contain a soft, largely unstructured liquid core that snaps onto the apolar silicon tip surface. These observations are in agreement with earlier empirical evidence noting that the core of LNPs lacks internal structure as the ionizable lipids become neutralized and is hypothesized to form a liquid oil phase at physiological pH.^5^

Following the negative force regime, a steep rise of force is seen in the force spectra, indicating that the tip reached the hard, incompressible glass substrate below the particle. The absence of constant-force transitions, characteristic of lipid (bi)layer breakthrough events,^18^ questions the presence of a phospholipid bilayer as an envelope. Upon retraction, initially, a negative force region is apparent that can be attributed to adhesive forces between the tip and the nanoparticle content. Following this stage, a region containing sawtooth-like transitions appeared in the force trace (marked by red asterisks in Figure 2A). The steady decrease in force (which corresponds to an increase in pulling force) is likely to reflect molecular strands (RNA) being pulled out from the particles, while the sudden force transitions are probably due to either the unfolding of RNA or the rupture of interactions between the RNA and lipid components (phases 3, 4). Alternative explanations could be that the AFM tip extracted supramolecular lipid assemblies from the NPs, referred as lipid tethers, or elongated the PEG moiety. However, the formation of lipid tethers typically yields force plateaus rather than force sawteeth,^19,20^ and the stretching of PEG chains could have caused shorter force sawteeth with a different shape.^21^

The superimposition of the retraction force traces (Figure 2C/1) failed to reveal preferred distances at which transitions occur. The number of force sawteeth ranged from a few (as seen in Figure 2A) to several (Figure 2C/2). The mean force associated with the sawtooth peaks was 42.7 pN (see peak force distribution in Figure 2D), which exceeds the force necessary for opening RNA hairpins (13–14 pN),^22^ suggesting that the observed transitions are the result of lipid–RNA interactions rather than RNA unfolding events. At the last transition, during which force returned to zero (i.e., no load on the cantilever), RNA was either pulled completely out of the particle or detached from the AFM tip (phase 5).

In control force spectroscopic measurements collected during indentation cycles performed on the background, force traces corresponding to wormlike chain (WLC) pulling were observed (Figure 2B). The persistence length calculated from the WLC fits is ∼0.3 nm, which is on the same scale as that of ssRNA.^23^ Lack of sawtooth-like transitions indicates no hairpin openings in RNAs being pulled from the supporting surface.

In sum, nanomechanical properties of NPs may play an important role in their cell adhesion and internalization, as it was demonstrated earlier for liposomes, extracellular vesicles, and viruses.^24−26^ The present study suggests that Comirnaty NPs are soft, deformable, and highly compliant structures.

### Cryo-TEM of Comirnaty

**Figure 3:**
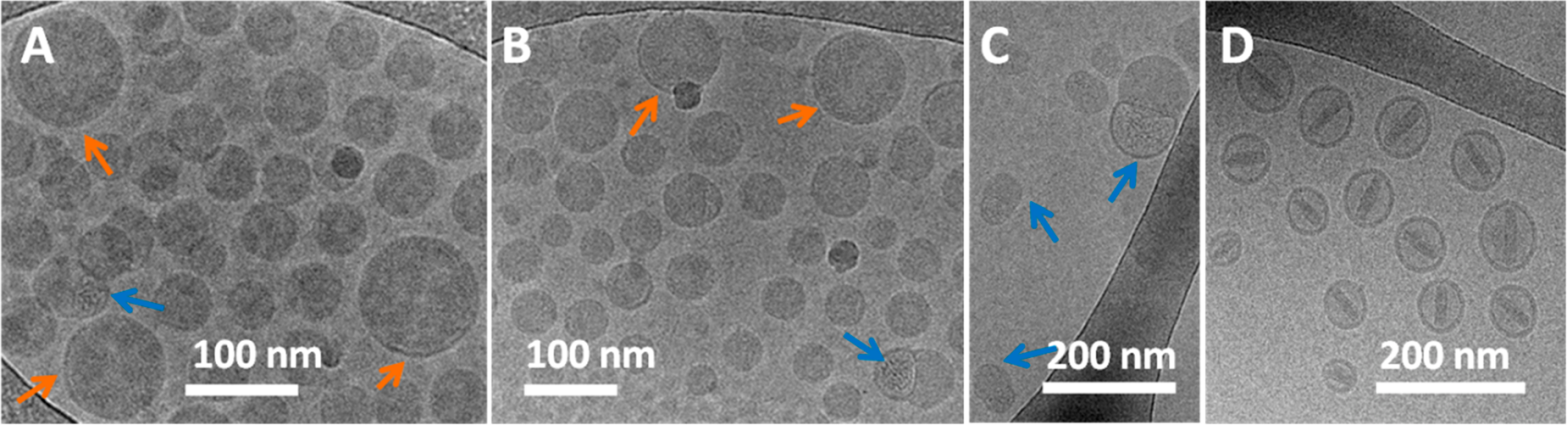
Cryo-TEM images of Comirnaty (A, C) and of Doxil (D). Comirnaty samples for cryo-TEM (A–C) were processed immediately after thawing the vaccine vial from −80 °C and dilution with saline, as instructed by the manufacturer for human application. Orange arrows show the bilayer coating of the NPs. Blue arrows point at bicompartmental NPs with an mRNA-containing bleb. (D) Cryo-TEM image of Doxil showing the rod-like doxoribicin-sulfate crystal inside the intra-liposome aqueous phase.

Consistent with the AFM images of fresh Comirnaty (Figure 1A) cryo-TEM images of the freshly diluted vaccine (Figure 3A–C) showed spherical NPs. These NPs exhibit a relatively broad size distribution having about 50 to 200 nm diameter. However, mostly, NPs having 50–80 nm diameters were observed, which may suggest a bimodal distribution. Many of the NPs have a solid core with an electron-dense interior, distinct from the diffused aqueous background. This appearance is similar to cryo-TEM images of LNPs filled with siRNA (with RNA-to-ionizable lipid ratio identical to Comirnaty)^5^ or mRNA (1.9 kb).^27^ Note that this interior is different from that in the anticancer nanodrug Doxil, where a low-density intra-liposome aqueous phase, similar to the background outside the liposome, is surrounding the nanorod-like doxorubicin-sulfate crystal inside the liposome (Figure 3D).

The cryo-TEM images of Comirnaty NPs highlight another phenomenon: the presence of electron-dense surface “caps” covering parts of the NPs (orange arrows), which resemble a bilayer coating, as in Doxil (Figure 3D). Thus, we hypothesize that these thick semicircle lines are phospholipid bilayers and that Comirnaty NPs are intermittently coated with phospholipid bilayers. In bilayer-free surface areas the IPC lipids and cholesterol should, of necessity, form monolayers.^5^ PEG lipids may also be present in these regions, as with lack of proper PEG surface coverage LNPs would quickly fuse.^27^ These observations are in keeping with the lack of clear signs of the lipid bilayer envelope by AFM force spectroscopy. Additionally, cryo-TEM images of all samples also included particles having a bicompartmental structure with a bilayer-coated bulge showing “grain-like” nanostructures related to mRNA (blue arrows in Figure 3A–C).^28^ It was suggested previously that these water-containing “blebs” may form upon segregation of DSPC from other lipids to form HSPC-enriched membranes/envelopes.^29^

### Negative Staining TEM of Comirnaty Enhances the Contrast of the Core Structure in Stored NPs

**Figure 4:**
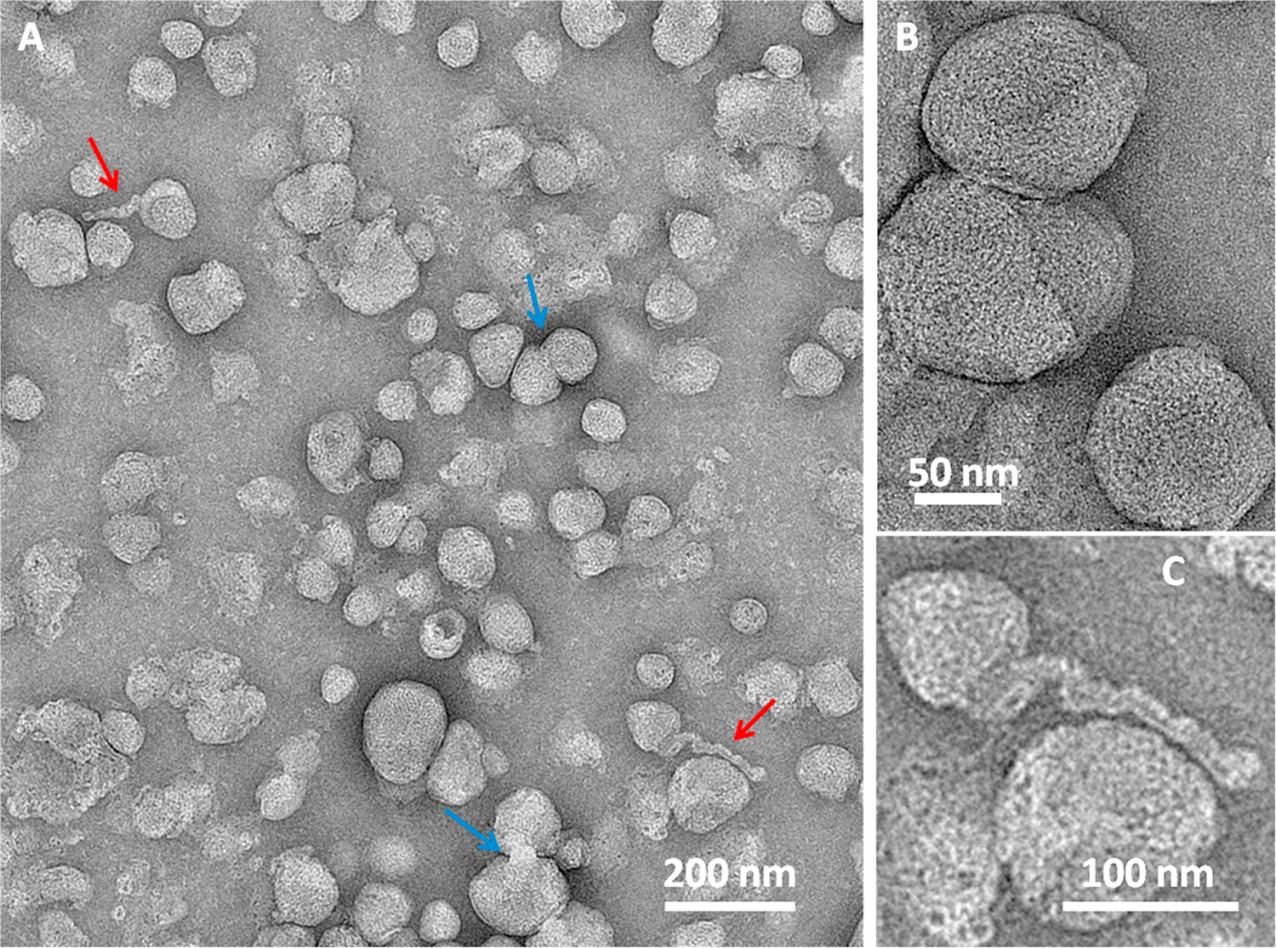
TEM images of a Comirnaty sample stored for 7 days in the refrigerator, diluted in water, and stained with acidic uranyl acetate as described in the Methods. Blue arrows in (A) point at the fusion of nanoparticles. (B) Higher magnification image of some LNPs. (C) Zoom-in of one of the elongated helical-like structures indicated by red arrows in (A).

The AFM force spectroscopy data suggested that mRNA is associated with lipids within the LNP core. This led us to hypothesize that the electron-dense gritty texture of LNP cores in cryo-TEM images (Figures 3A, B) reflects lipid-bound, condensed state mRNA mingled with the excess of ionizable lipids. This hypothesis was further supported by TEM of a sample stored for a week in the refrigerator and then subjected to negative staining with pH 4.5 uranyl acetate. This dried and stained sample (Figure 4) showed a wide variety of odd-shaped LNPs and some molecular details not seen with cryo-TEM. Higher magnification of some of the globular structures (Figure 4B) shows circular and semicircular chains of ∼2–3 nm dots lined up in balls of yarn-like lumps. These may be rationalized as mRNA “supercoils” held tightly together by intra- and interchain forces. Zooming into some structures (red arrows) in Figure 4A, we see intertwined helices winding out from NP remains, which are probably mRNA-containing fragments (Figure 4C), while other structures seem to capture the process of fusion (Figure 4A, blue arrows).

Although the above transitional structures could be artifacts in the dried and negatively stained LNPs, breakup and fusion of NPs were also seen in the 1 day stored AFM images (Figure 1B), and the possibility of mRNA chains leaving the LNP was also suggested by the stepwise pulling out of string-like structures from Comirnaty by the AFM tip (Figure 2). Furthermore, these fragmented nanostructures were seen in samples stained with acidic uranyl acetate, i.e., under conditions known to lead to the disintegration of mRNA-LNPs in the acidic milieu of endosomes, the site of mRNA release into the cytosol.^30^ Thus, while exposing unforeseen details of LNP core structure, these transient NPs may illustrate the intra-endosomal transformation of LNPs after vaccination, in vivo.

Beyond the acidic milieu, the other likely contributing factor to LNP disintegration is the limited stability of the vaccine in water, as reflected in the brief (up to 6 h) shelf life of Comirnaty at RT after dilution.^31,32^

### Comparing the Transmembrane pH Gradient of Comirnaty and Doxil

Transmembrane ion and pH gradients are measures of NP membrane ability to maintain intra-liposome ion (including protons) concentration. Changes and stability of intra-NP proton concentration can be determined for the pH gradient (NP pH ≪ medium pH).^33^ The larger the pH gradient, the higher the accumulation of ^14^C-MA in the NPs.^34^ Therefore, we compared the pH gradients in Comirnaty and Doxil, Myocet (nonPEGylated liposomal doxorubicin), and Marqibo (nonPEGylated liposomal vincristine). In all three liposomal drugs, the drug encapsulation is driven by transmembrane pH gradients. In Doxil, this is achieved by the use of transmembrane ammonium sulfate gradient,^35^ while for Myocet^36^ and Marqibo,^36^ the pH gradient is ensured by preparing the LNP in citrate buffer, pH 4.0, and raising the pH to neutral (pH 7.2) after the active ingredient is encapsulated.

Measurement of the pH gradient in Comirnaty was performed by measuring the ^14^C methylamine (^14^C-MA) distribution in Comirnaty and Doxil. This is especially relevant to Comirnaty LNPs, as their active ingredient (mRNA) encapsulation is done in a similar exposure to medium pHs, starting at a pH 4.0, followed by changing the medium pH to neutral (pH 7.2).

For all Comirnaty samples, regardless of the storage time, there was no ^14^C-MA accumulation by the LNPs, which indicates no pH gradient between the LNP core and the external medium. In contrast, for Doxil, which has a similar envelop lipid composition to Comirnaty (DSPC, the main component of HSPC, cholesterol, and PEGylated lipid), the measured ΔpH is 1.8 and the calculated internal pH is 4.7.^33^ Similar pH gradients were determined for Myocet and Marqibo.^36^

The loss of internal acidic pH has a fundamental impact on the level of ionization of IPC lipid in Comirnaty. ALC 0315 has a p*K*_a_ of 6.5 and therefore it has a strong cationic character only during LNP formation at pH 4.0.^37,38^ The rise of intra-LNP pH entails decreased positivity of IPC lipids and thus weakening of their ionic interactions with the mRNA. This could contribute to the gradual disintegration of LNPs after dilution and storage and explains the softness and fragility of Comirnaty NPs after dilution. The increased ion permeability of Comirnaty NPs could be due, at least in part, to the lack of continuous surface bilayer (Figure 3A, B), since Doxil, which has a similar envelope lipid composition but in the form of a continuous bilayer, does not lose the pH gradient in vitro during storage as a liposome dispersion and *in vivo*.^35,39^

### Current LNP Models May Not Be Applicable to Comirnaty

**Figure 5:**
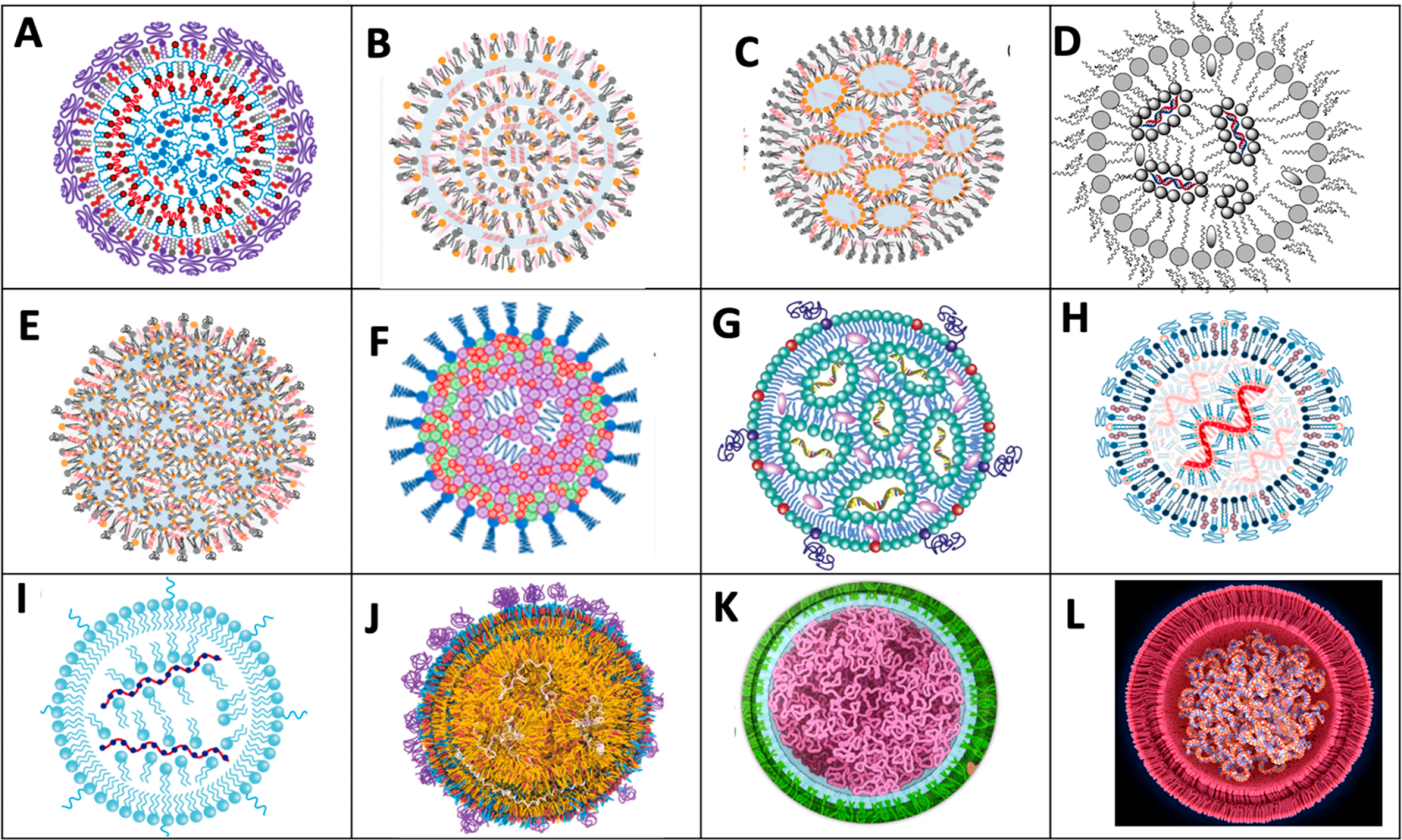
Schematic current models of nucleic acid containing LNPs illustrating the variety of concepts. (A, B) “Multilamellar vesicle model” with siRNA sandwiched among the bilayers.^5,40^ In (A) there is an IPC lipid/cholesterol core,^5^ while in (B), the lipids are not differentiated. (C) Phospholipid monolayer-covered inverted micelle core.^41^ The siRNA is shown within the surface monolayer and intermicellar space.^41^ (D) Phospholipid-monolayer-coated inverted IPC micelles with siRNA inside the aqueous core of micelles.^42^ (E) Phospholipid-monolayer-coated inverted micelles with the siRNA randomly distributed.^41^ (F) PEG-lipid-coated random assembly of lipids and siRNA.^43^ (G) Phospholipid-monolayer-coated assembly of inverted IPC lipids. The mRNA is attached to the inner layer of inverted micelles.^44^ (H) Phospholipid-bilayer-coated assembly of mRNA, covered with IPC lipids.^45^ (I) Same as (H), except that the outer membrane is a monolayer.^46^ (J) mRNA randomly distributed in an amorphous lipid matrix with heterogeneous bilayer surface coat.^47^ (K) mRNA thread ball with no identifiable lipid and membrane components. (L) Bilayer-coated mRNA thread ball with no identifiable lipids.^48^

There are many features of Comirnaty that cannot be reconciled with the current models of the LNP structure developed for small interfering RNAs (siRNAs). In one of these models, referred to as the “multilamellar vesicle model”, the 7–8 nm, 21–23 nucleotide-containing siRNA polynucleotides were proposed to be sandwiched between tightly packed IPC-enriched monolayers and/or bilayers (Figure 5A, B). However, such a particle structure should result in distinct force transitions (bilayer breakthroughs) upon NP indentation with the AFM’s cantilever, which was not observed (Figure 2).

In another concept, known as “core–shell” or “nanostructured core” model, the phospholipid monolayer-covered LNPs contain water-filled inverted micelles (Figure 5C) and the siRNA molecules are in the intermicellar space and within the outer monolayer membrane. In an alternative of the latter model, the siRNA molecules are located only in the intermicellar space (Figure 5D). Figure 5E and F show further models in which the relative positions of lipids and siRNA are vague.

As for mRNA LNPs, such as Comirnaty, one must consider that the ∼1414 kDa, 4284 nucleotide-containing mRNA in Comirnaty has an extended length of ∼1500 nm, which is roughly 180 times longer than a siRNA. This huge size difference between siRNA and mRNA has been well recognized,^49^ yet this dissimilarity is not illustrated in most schematic cartoons of mRNA-LNP models. For example, in one, the minimally larger mRNA (compared to siRNA) is shown as being bound to the charged inner layer of inverted micelles made of IPC (Figure 5G) or as spirals covered by IPC lipids (Figure 5H, I). Yet another model of mRNA-LNP shows randomly distributed mRNA in an amorphous lipid core. The envelope of mRNA-LNPs is variably shown as a phospholipid-enriched monolayer (Figure 5G, I) or bilayer (Figure 5H).

Taken together, visual representations of mRNA-LNP models are still far from being to scale; the lamellarity of the outer membrane is ambiguous, and the relationship between the mRNA and lipids is highly variable in the different models. These facts highlight the need to better understand the molecular structure of mRNA-LNPs and accordingly better visualize them in schematic models.

### Proposal of an LNP Model Specific for Comirnaty

According to Buschmann et al., each Comirnaty LNP contains up to 10 mRNA molecules.^40^ Considering the above-mentioned size of Comirnaty mRNA, they cannot fit into 60–150 nm diameter spheres unless they tightly wind in dense loops or supercoil-like structures, just as the DNA fills the nuclei. There exist such vaccine presentations in the public media, showing the mRNA in the vaccine LNPs as balls of yarn or thread balls (Figure 5K, L). However, these simplified, fictional models make no attempt to illustrate the details of the membranes and the relationships among the different internal components, most critically the IPC lipids, whose number exceeds that of the mRNA nucleotides 6-fold.^50^

**Figure 6:**
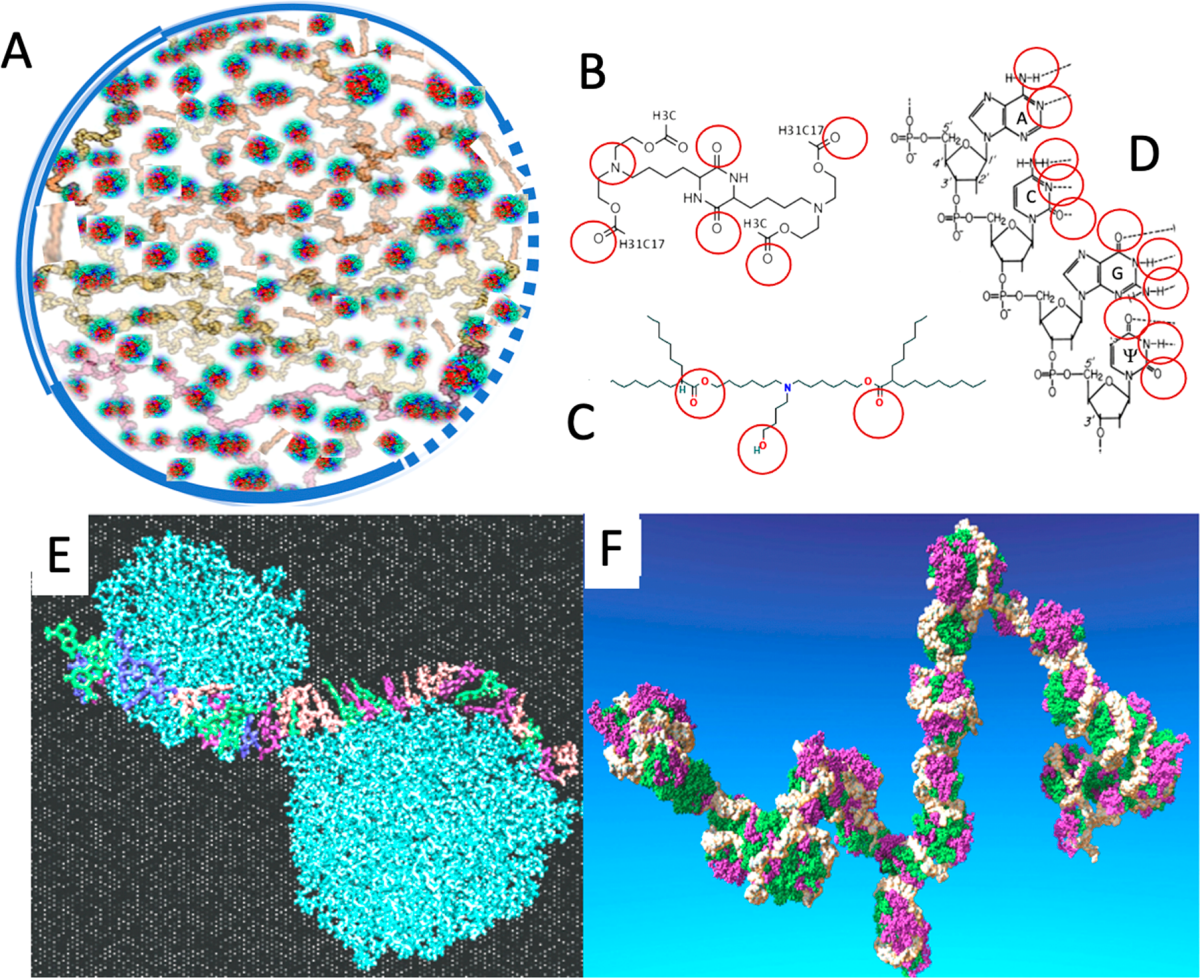
(A) Scheme of a Comirnaty-specific LNP model wherein the stability of yarn ball-like mRNA lumps (gray) is secured by IPC lipid cross-links and clusters (green and red dots). The NP is surrounded by a phospholipid bilayer (double line), monolayer (single line), or no membrane (dotted line). (B) Chemical structure of DML, containing a diketopiperazine core, two methyl ester end-groups, and two linoleic ethyl ester end-groups.^7^ (C) Chemical structure of ALC-0315, the ionizable cationic lipid in Comirnaty [(4-hydroxybutyl)azanediyl) bis(hexane-6,1-diyl) bis(2-hexyldecanoate (Table S1 and ref (50)). (D) A four-nucleotide section of the mRNA chain in Comirnaty showing the N and O atoms available for hydrogen bonding, ψ, pseudouridine. (E) The Rissanau et al. model of DML lipid cluster–mRNA complexation shows a 30-nucleotide-containing short mRNA meandering along 2 DML clusters. (F) Molecular dynamics simulation of a 642-nucleotide-containing mRNA complexed with multiple DML clusters that protect the mRNA from hydrolysis. (E) and (F) are reprinted from ref (7) with permission from the Royal Society of Chemistry.

The analytic and visual data in the present study, taken together with the mentioned size information, suggest a hypothetical model wherein a single or multiple copies of mRNA-IPC complexes densely fill up part or the whole internal space of LNPs. The IPC lipids, or self-associated IPC clusters, stabilize the tertiary structure of mRNA via intra- and interloop cross-links and bridges (Figure 6A). Cross-links may be provided by hydrogen bonds between the free =O and −OH groups of IPC lipids and the nitrogenous bases in the mRNA, while bridges may be formed from IPC clusters.^7^

The stability of RNA–lipid clusters may be secured in the frozen vaccine by the fact that the molecules do not vibrate as vigorously as in the unfrozen state,^51^ and it may be increased by electrostatic interactions with IPC lipids as a function of their positivity. However, both the hydrogen bonds, which are considered to be weak bonds, and the electrostatic bonds, whatever still exists at neutral (7.2) pH, become weaker as the temperature increases, as occurs during storage of the aqueous dispersion at RT, and even faster when the mRNA-LNP reaches the temperature of body fluids (37 °C).

The above weakly stabilized “mRNA-based lipoplex” model of Comirnaty mRNA-LNP was inspired by a recent study by Rissanou et al.^7^ showing that an IPC lipid (called DML, details are seen in the legend of Figure 6), which is very similar to Comirnaty’s IPC (ALC 0315) in terms of molecular size and steric positions of free OH– and O= groups, can theoretically align with similar groups on the purine and pyrimidine bases of single stranded mRNA (red circles in Figure 6B).

For DML, evidence was provided that it can self-associate into clusters that can bind to mRNA by both ionic and hydrogen bonds,^7^ and thus stabilize the tertiary structure of the complex in a molecular assembly where the mRNA meanders around lipid clusters (Figure 6E, F). We hypothesize that ALC 0315 in Comirnaty may have similar mRNA binding capability and stabilize Comirnaty’s mRNA in the delineated condensed conformation, in a supercoil-like, glomerular lattice structure.

## Conclusions

One insight into the nanostructure of the Comirnaty Covid-19 vaccine concerns the tertiary structure of mRNA within the LNPs and its interaction with IPC lipids. Our experimental data and theoretical considerations suggest that the highly convoluted mRNA strains are condensed in the core of NPs primarily by temperature-dependent weak inter- and intramolecular interactions, involving hydrogen bonds and, depending on the actual pH, ionic bonds with IPC lipids. However, ionic interactions between the nucleic acid and IPC lipids may be insignificant or absent at neutral pH in the vaccine’s saline-diluted formulation for human use. The predominance of hydrogen bonds and lack of a continuous bilayer around the mRNA–lipid matrix offer an explanation for the soft and compliant structure of Comirnaty LNPs, as well as the vaccine’s limited stability in ambient temperature.

Another insight, visualization of breakup products of mRNA-LNPs in the negatively stained TEM samples of stored vaccine, provides glimpses into the likely “destiny” of Comirnaty NPs in acidic endosomes.

Despite its softness and limited stability, the vaccine is clearly very efficacious in inducing an immune response, raising the possibility that the above highlighted structural features are contributing to or may be essential for its immunogenic function. Our data may therefore lay the grounds for a paradigm shift in understanding the structure–function relationship in mRNA-LNP-based vaccines and other therapeutics.

## Methods/Experimental

### Materials

BNT162b2 (Comirnaty) was from Pfizer/BioNtech, a vaccine used for human vaccinations against SARS-CoV-2 infections. The frozen vials were thawed and then diluted with saline by a healthcare professional as instructed by the manufacturer. Doxil was obtained from a local pharmacy. The compositions of the vaccine and Doxil are tabulated in Supplementary Table S1.

### AFM Imaging and Force Spectroscopy

For AFM experiments, original, sterilized, unexpired batches of Comirnaty were diluted in physiological salt solution. The diluted sample was used immediately. Further experiments were done with samples (a) stored at 4 °C for 1 day or (b) frozen in liquid N_2_, stored at −20 °C for 1 day, and thawed to room temperature. Round coverslips (Ted Pella Inc., Redding, CA, USA) were glued with UV epoxy onto metal AFM specimen disks and cleaned with ethanol and then methanol. The cleaned glass surface was dried in a nitrogen stream. A 100 μL aliquot of vaccine was dropped onto the freshly cleaned surface and incubated for 20 min at room temperature. Then the surface was washed 5 times with 100 μL of PBS (16 mM Na_2_PO_4_, 4 mM NaHPO_4_, 150 mM NaCl; pH 7.4). Imaging was carried out in tapping mode at 25 °C with a Cypher ES AFM (Asylum Research, Santa Barbara, CA, USA) using a BL-AC40TS cantilever (silicon nitride, nominal stiffness: 90 pN/nm, tip radius: 8 nm; Olympus, Japan) at typical line rates of 0.5 Hz. The cantilever was oscillated photothermally (BlueDrive) at its resonance frequency (typically 20 kHz in water). Prior to the measurements, the cantilevers were calibrated by using the thermal method in air.^52^

In situ force spectroscopy was carried out in contact mode on vesicles selected from a previously scanned AFM image. During force spectroscopy, the cantilever was moved vertically with a speed of 1 μm/s from a height of 500 nm toward the vesicle vertex until a force threshold of 5 nN was reached. Then the tip was immediately retracted at the same speed. Deflection of the cantilever, hence force, as a function of cantilever position (force–indentation curve or force curve) was recorded during the process. Images and force spectroscopy data were analyzed by using the built-in algorithms of the AFM driving software (IgorPro, WaveMetrics Inc., Lake Oswego, OR, USA).

### Measurement of Comirnaty NP Size and Size Distribution by Dynamic Light Scattering

DLS measurements were performed immediately after thawing a Comirnaty vial and diluting NPs with saline (performed by a healthcare professional). Size (diameter, *D*), size distribution (polydispersity index, PDI, and SPAN), and zeta potential (Zp) of the NPs were determined with a Malvern Zetasizer Nano ZS instrument (Malvern, Worcestershire, UK) at an angle of 173°. In addition to the PDI, a standard measure of homogeneity, we also determined the SPAN, a better measure of distribution broadness than PDI. It is defined as *D*_90_ – *D*_10_/*D*_50_ where *D*_10, 50, and 90_ are the percentiles under the size distribution curve.

### TEM and Cryo-TEM Imaging

Negative-staining transmission electron microscopy (TEM) was performed by applying a drop (3 μL) of sample to a glow-discharged TEM grid (carbon-supported film on 300-mesh Cu grids, Ted Pella, Ltd.). After 30 s the excess liquid was blotted, and the grids were stained with 2% uranyl acetate for 30 s and allowed to dry in air. Imaging was carried out using a FEI Tecnai 12 G2 Twin TEM operated at 120 kV. The images were recorded by a 4Kx4K FEI Eagle CCD camera using the TIA software. Direct imaging of samples by cryo-TEM was performed as described elsewhere.^39^ A 3 μL sample was applied onto a glow-discharged 300-mesh copper TEM grid coated with a holey carbon film (Lacey substrate, Ted Pella, Ltd.). The excess liquid was blotted and the specimens were vitrified by rapid plunging into liquid ethane precooled by liquid nitrogen using a Vitrobot Mark IV (FEI). We then transferred the vitrified samples into a cryo specimen holder (Gatan model 626; Gatan Inc.) and imaged them at −177 °C using a Tecnai 12 G2 Twin TEM (FEI), operated at an acceleration voltage of 120 kV in low-dose mode. Images were recorded with a 4Kx4K FEI Eagle CCD camera. TIA (Tecnai Imaging & Analysis) software was used to record the images.

### Measurement of Intra-LNP pH/pH Gradient

The assay, originally described by Abraham et al.,^34^ consisted of measuring the distribution of radioactive methylamine (^14^C-MA) between the NPs and solvent medium, as applied earlier for Doxil.^33^ In brief, ^14^C-MA was added to freshly diluted Comirnaty LNPs. After 20 min of incubation at 55 °C, the LNP dispersion was divided equally into two parts. One part of the LNP solution was centrifuged using Amicon Ultra-15 tubes using a filter of 100 K molecular weight cutoff (Millipore, MA, USA) for the separation of the unencapsulated (extra-LNP) from the encapsulated ^14^C-MA. The medium collected after centrifugation and the other part of the original liposome dispersion (before centrifugation) were first treated with Opti-Fluor (PerkinElmer, Waltham, MA, USA) and stored overnight at 2–8 °C before scintillation counting of the radioactivity by a β-counter. The ratio of ^14^C-MA between the LNPs and the extra-LNP medium was used to calculate the transmembrane pH gradient as follows: ΔpH = log{[H^+^]inside/[H^+^]outside = log{[methylamine] inside/[methylamine] outside.

## Data Availability

Source data are provided with this paper and its Supplementary Table 1. All other data supporting the findings of this study are available from the corresponding author upon reasonable request.

## Supporting Information Available

Compositions and other characteristics of Comirnaty and Doxil (PDF)

## Notes

Preprint: János Szebeni, Bálint Kiss, Tamás Bozó, Keren Turjeman, Yael Levi-Kalisman, Yechezkel Barenholz, Miklós Kellermayer: New insights into the structure of Comirnaty Covid-19 vaccine: A theory on soft nanoparticles with mRNA-lipid supercoils stabilized by hydrogen bonds. https://www.biorxiv.org/content/10.1101/2022.12.02.518611v1. Posted: December 05, 2022.
