# Supplementary material for: Insights into the Structure of Comirnaty Covid-19 Vaccine: A Theory on Soft, Partially Bilayer-Covered Nanoparticles with Hydrogen Bond-Stabilized mRNA–Lipid Complexes
Source: ACS Nano. 2023 Jul 7;17(14):13147–57. doi: 10.1021/acsnano.2c11904 (PMC10373524; doi:10.1021/acsnano.2c11904)
Supplement: Supplementary file 1 — nn2c11904_si_001.pdf [file nn2c11904_si_001.pdf]

Supplemental Information

Insights into the structure of Comirnaty® Covid-19 vaccine: A theory on soft, partially bilayer-covered nanoparticles with hydrogen bond-stabilized mRNA-lipid complexes

János Szebeni,[1-3,#,*] Bálint Kiss,[4,5,6] Tamás Bozó,[4,5] Keren Turjeman,[7] Yael Levi-Kalisman,[8] Yechezkel Barenholz[7#], Miklós Kellermayer[4,5,6#]

Affiliations:

1. Nanomedicine Research and Education Center, Department of Translational Medicine, Semmelweis University, Budapest 1089, Hungary
2. Department of Nanobiotechnology and Regenerative Medicine, Faculty of Health Sciences, Miskolc University, Miskolc 2880, Hungary
3. School of Chemical Engineering and Translational Nanobioscience Research Center, Sungkyunkwan University, Suwon 16419, Korea
4. Department of Biophysics and Radiation Biology, Semmelweis University, Budapest 1094, Hungary
5. Hungarian Centre of Excellence for Molecular Medicine (HCEMM), In Vivo Imaging Advanced Core Facility, Budapest 1094, Hungary
6. ELKH-SE Biophysical Virology Research Group, Tűzoltó Str. 37-47, Budapest 1094, Hungary
7. The laboratory of Membrane and Liposome Research, IMRIC, Hebrew University-Hadassah Medical School, Jerusalem 9112102, Israel
8. Institute of Life Sciences, and the Center for Nanoscience and Nanotechnology, The Hebrew University of Jerusalem, Edmond J. Safra Campus, Givat Ram, Jerusalem, 9190401, Israel

Table S1. Composition and other characteristics of Comirnaty and Doxil

Nano-formulation	Component	Molecular formula	MW (gr/mol)	Structure	Packing parameter[a]	Concentration (mg/ml)	Concentration (mM)	Mole%	Total lipids (mg/ml)
**Comirnaty® (Pfizer-BioNTech BNT162b2 mRNA-LNP vaccine)**	Cationic lipid (ALC-0315)	$C_{48}H_{95}NO_5$	766.3	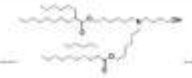	>1.0	1.4	1.87	46.1	2.6
	PEGylated lipid (ALC-0159)	$(C_2H_4O)nC_{31}H_{63}NO_2$	2330	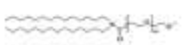	~0.5	0.2	0.07	1.8	
	DSPC	$C_{44}H_{88}NO_8P$	790.1	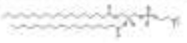	0.8	0.3	0.38	9.4	
	Cholesterol	$C_{27}H_{46}O$	386.6	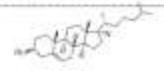	1.2	0.7	1.73	42.8	
	mRNA (per nucleotide)		333		NR	0.1	0.30	-	
**Doxil®**	DSPE-PEG (2000)	$C_{45}H_{86}NO_{11}P$	2700	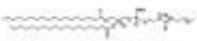	~0.5	3.2	1.19	5.4	16
	HSPC	$C_{42}H_{84}NO_8P$	788	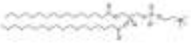	0.8	9.6	12.18	56.3	
	Cholesterol	$C_{27}H_{46}O$	386	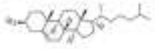	1.2	3.2	8.29	38.3	
	Doxorubicin	$C_{27}H_{29}NO_{11}$	580	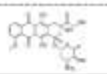	NR	2.0	3.45	-	

[a]Estimation based on structure and Garbuzenko et al.[1]

1. Garbuzenko, O.; Zalipsky, S.; Qazen, M.; Barenholz, Y., Electrostatics of PEGylated micelles and liposomes containing charged and neutral lipopolymers. *Langmuir* 2005, *21*, 2560-256
